# PANoptosis and mitochondrial regulatory mechanisms in cerebral ischemia-reperfusion injury

**DOI:** 10.3389/fphys.2026.1759575

**Published:** 2026-03-06

**Authors:** Li Li, Chunyan Guo, Zheng Zuo, Luoyang Cai, Xin Chen, Yongjiang Fang, Shengnan Zhang, Tianyu Chen, Peng Kuang, Pengyue Zhang, Li Li, Zuhong Wang

**Affiliations:** 1 Yunnan University of Chinese Medicine, Kunming, Yunnan, China; 2 The Third Affiliated Hospital of Yunnan University of Chinese Medicine, Kunming, Yunnan, China; 3 Yunnan Cancer Hospital, Kunming, Yunnan, China

**Keywords:** cell death pathways, cerebral ischemia-reperfusion injury, mitochondrial dysfunction, neuroinflammation, neuroprotection, PANoptosis

## Abstract

Cerebral ischemia-reperfusion injury remains a leading cause of mortality and disability despite advances in reperfusion therapy. Traditional research has focused on individual cell death pathways, yet pharmacological blockade of single pathways provides only partial neuroprotection, suggesting that dying cells engage multiple death routes simultaneously. This review examines whether PANoptosis, an inflammatory cell death modality characterized by concurrent activation of apoptotic, necroptotic, and pyroptotic pathways, occurs in cerebral ischemia-reperfusion injury. The analysis demonstrates that mitochondrial dysfunction serves as the central convergence point orchestrating multi-pathway death activation across distinct temporal phases. Ischemia creates metabolic crisis that primes mitochondria without triggering irreversible commitment. Reperfusion causes explosive mitochondrial collapse through oxidative stress, releasing danger signals that simultaneously engage multiple death pathways. Impaired mitochondrial quality control then sustains inflammatory amplification over extended periods. Multiple lines of evidence support this framework, including concurrent rather than sequential appearance of pathway markers, mixed morphological features within individual cells, pathway redundancy demonstrated by incomplete single-target protection, and mechanistic convergence at the mitochondrial level. Cellular responses vary among neurons, astrocytes, microglia, and endothelial cells but share the common feature of coordinated multi-pathway activation. This integrated understanding explains why single-pathway therapeutic approaches have failed clinically and suggests that effective neuroprotection requires targeting upstream mitochondrial dysfunction or addressing pathway redundancy through multi-target interventions.

## 1 Introduction

Cerebral ischemia-reperfusion injury represents a major cause of mortality and long-term disability worldwide (Feigin et al., 2024). Ischemic stroke occurs when arterial occlusion interrupts blood flow to brain tissue and deprives neurons of oxygen and glucose required for survival (Stoll et al., 2024). Although modern reperfusion therapies including thrombolysis and thrombectomy can restore blood flow, clinical outcomes remain suboptimal and many patients develop substantial neurological deficits despite successful recanalization (Mendelson and Prabhakaran, 2021; Volpe and Patrono, 2022; Xiong et al., 2022; Widimsky et al., 2023). The injury extends beyond the brief period of blood flow interruption, as restoration of circulation paradoxically triggers additional pathological cascades involving metabolic failure, oxidative stress, inflammatory activation, and widespread cell death (Fricker et al., 2018; Tuo et al., 2022). Understanding the mechanisms governing cell death in cerebral ischemia-reperfusion injury holds importance for developing neuroprotective strategies that might improve patient outcomes.

Traditional research investigating cell death in cerebral ischemia-reperfusion injury has focused on individual death modalities, examining apoptosis, necroptosis, or pyroptosis as separate processes (Naito et al., 2020; Xia et al., 2020; Long et al., 2023; Truong et al., 2023; Li L. et al., 2024; Mei et al., 2025). However, accumulating evidence reveals that these pathways do not operate in isolation. Dying neurons frequently exhibit mixed morphological features combining characteristics of multiple death modalities (Hollville et al., 2019; Jones, 2020). Pharmacological or genetic blockade of individual pathways provides only partial neuroprotection, suggesting that alternative death routes remain functional when one pathway is inhibited (Ismael et al., 2018; Deng et al., 2019). These observations indicate that the framework of independent death pathways inadequately explains the complex cell death patterns observed in ischemic brain tissue. The PANoptosis concept, which describes inflammatory cell death characterized by concurrent engagement of apoptotic, necroptotic, and pyroptotic machinery, emerged primarily from infectious disease and inflammatory research (Li, 2021; Sundaram et al., 2024). Whether this concept applies to cerebral ischemia-reperfusion injury, a condition driven by metabolic stress rather than pathogen detection, remains an open question that this review addresses.

Mitochondria warrant particular emphasis because they occupy a central position in both cerebral ischemia pathology and death pathway regulation (Tang et al., 2016). Blood flow interruption immediately halts mitochondrial respiration and initiates a bioenergetic crisis. Beyond their metabolic function, mitochondria also serve as integrators and amplifiers of death signals through multiple mechanisms including outer membrane permeabilization, mitochondrial DNA release, reactive oxygen species production, calcium dysregulation, dynamics imbalance, and quality control failure (Chinnery et al., 2010; Liu D.-Q. et al., 2024). These mitochondrial dysfunctions interconnect through positive feedback loops that amplify pathology. If PANoptosis occurs in cerebral ischemia-reperfusion injury, mitochondria likely serve as the nexus where death pathway activation is coordinated. The unique temporal dynamics of cerebral ischemia-reperfusion injury create distinct phases where mitochondrial dysfunction manifests differently. Ischemia primes mitochondria without necessarily triggering irreversible death. Reperfusion causes explosive mitochondrial collapse that activates multiple death pathways simultaneously. Failed quality control allows damaged mitochondria to accumulate and drive sustained inflammatory amplification over days to weeks.

The observation that dying cells in cerebral ischemia-reperfusion injury exhibit markers of multiple death pathways raises the question of whether this reflects PANoptosis or simply parallel activation of independent pathways. This review evaluates available evidence to address this question by examining how mitochondrial dysfunction might orchestrate coordinated multi-pathway death activation across the temporal phases of cerebral ischemia-reperfusion injury. The analysis integrates mechanisms with their pathological manifestations to show how ischemic priming, reperfusion collapse, and failed quality control create a progression from metabolic crisis to multi-pathway death activation to sustained inflammatory amplification. Four categories of evidence are evaluated to determine whether the PANoptosis framework applies to this condition. These include temporal co-occurrence of pathway markers, mixed cellular morphology, pathway redundancy demonstrated by incomplete protection from single-target interventions, and mechanistic convergence at the mitochondrial level. The review addresses cellular heterogeneity among neurons, astrocytes, microglia, and endothelial cells throughout this temporal framework. Understanding whether mitochondrial dysfunction coordinates multi-pathway cell death through integrated or independent mechanisms provides a framework for developing neuroprotective strategies that address pathway redundancy rather than targeting individual death modalities, potentially explaining why single-pathway approaches have repeatedly failed in clinical translation despite preclinical promise.

## 2 PANoptosis: conceptual framework and molecular features

### 2.1 Evolution of the PANoptosis concept

The understanding of programmed cell death has undergone substantial transformation over the past decades (Amaral et al., 2020). Initially, apoptosis, necroptosis, and pyroptosis were conceptualized as distinct and independent death programs, each characterized by unique morphological features, biochemical signatures, and molecular executioners. Apoptosis was defined by nuclear condensation, DNA fragmentation, and plasma membrane integrity maintenance (Shi et al., 2017). Necroptosis exhibited cell swelling, membrane rupture, and receptor-interacting protein kinase 1/3 (RIPK1/3) activation (Hu X. et al., 2022). Pyroptosis was distinguished by gasdermin family protein-mediated membrane pore formation and robust inflammatory cytokine release (Yu P. et al., 2021). This compartmentalized view provided a useful framework for studying individual death pathways but proved insufficient for explaining the complexity observed in pathological conditions.

Accumulating evidence revealed extensive molecular crosstalk between these supposedly independent pathways. Key regulatory proteins were found to participate in multiple death programs. Caspase-8, traditionally considered an apoptotic initiator, was discovered to suppress necroptosis by cleaving RIPK1 and RIPK3 (Newton et al., 2019; Someda et al., 2020). Conversely, when caspase activity was compromised, cells could switch from apoptosis to necroptosis (Fritsch et al., 2019). Similarly, inflammasome activation could simultaneously trigger pyroptotic and apoptotic cascades under certain conditions (Zheng and Kanneganti, 2020; Huang et al., 2021). These observations challenged the rigid boundaries between death modalities and suggested a more integrated regulatory network. The realization emerged that under specific pathological circumstances, particularly during infection and intense inflammatory stimulation, cells could simultaneously activate multiple death pathways rather than committing to a single program.

This multi-pathway activation phenomenon prompted Kanneganti and colleagues to propose the PANoptosis concept in 2019 (Malireddi et al., 2019). PANoptosis describes a lytic inflammatory cell death pathway characterized by concurrent activation of key molecules from apoptotic, necroptotic, and pyroptotic pathways, executed through formation of a multi-protein complex termed the PANoptosome. Rather than representing mere co-occurrence of independent pathways, the PANoptosome integrates proteins from distinct death programs into a single assembled complex, enabling coordinated activation of caspase-1, caspase-8, caspase-3, and mixed lineage kinase domain-like protein (MLKL), the respective executioners of pyroptosis, apoptosis, and necroptosis (Jadhav et al., 2024).

**FIGURE 1:**
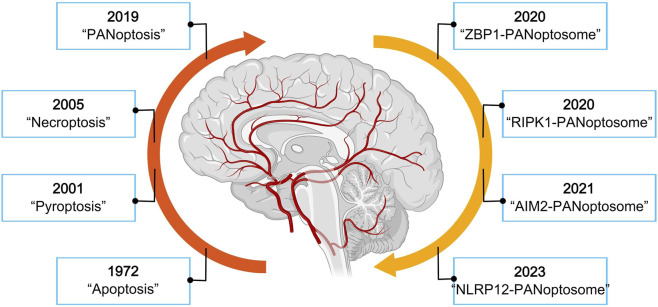
Timeline of cell death research evolution. This figure is created with MedPeer (medpeer.cn).

The PANoptosis concept remains under active refinement, and discussions continue regarding its precise definition and boundaries. The development of PANoptosis as a distinct cell death modality reflects decades of accumulated understanding regarding regulated cell death mechanisms (Figure 1). Most research demonstrating PANoptosis has been conducted in infection models and inflammatory contexts involving immune cells (Karki et al., 2021; Lee et al., 2021). Whether PANoptosis applies to cerebral ischemia-reperfusion injury, a condition driven by metabolic stress and oxidative damage rather than pathogen recognition, requires systematic evaluation of the available evidence.

### 2.2 Molecular composition and activation features of the PANoptosome

To elucidate the molecular basis of PANoptosis, Christgen and colleagues proposed the PANoptosome concept in 2020, defining it as the large multiprotein complex that drives PANoptosis (Christgen et al., 2020). The PANoptosome integrates key molecules from different death pathways, serving as a molecular platform for multi-pathway activation. Early studies of TAK1-deficient cells revealed that a protein complex containing RIPK1, apoptosis-associated speck-like protein containing a caspase recruitment domain (ASC), NLRP3, and caspase-8 could simultaneously activate multiple death pathways (Malireddi et al., 2020). Subsequent research gradually unveiled the complete composition and different subtypes of the PANoptosome.

Currently identified PANoptosomes include the ZBP1-PANoptosome, AIM2-PANoptosome, RIPK1-PANoptosome, and NLRP12-PANoptosome (Table 1). The ZBP1-PANoptosome was the earliest comprehensively characterized type, primarily responding to viral infections such as influenza virus. Studies showed that ZBP1 recognizes Z-DNA or Z-RNA through its Zα domain and interacts with RIPK3 via its RHIM domain, recruiting ASC, NLRP3, caspase-1, caspase-8, and RIPK1 to form the complex (Kesavardhana et al., 2020). The AIM2-PANoptosome plays important roles during herpes simplex virus and Francisella novicida infections. Following cytosolic DNA recognition, AIM2 not only activates the classical inflammasome pathway but also regulates the functions of ZBP1 and pyrin, forming a large complex containing ASC, caspase-1, caspase-8, RIPK3, RIPK1, and Fas-associated death domain protein (FADD) (Lee et al., 2021). The RIPK1-PANoptosome was discovered during *Yersinia* pseudotuberculosis infection, characterized by RIPK1 forming immunoprecipitation complexes with RIPK3, caspase-8, ASC, FADD, and NLRP3 (Polykratis et al., 2014).

**TABLE 1:** Molecular composition and activation characteristics of PANoptosome subtypes.

PANoptosome subtype	Sensor molecules	Primary triggers	Representative contexts	Ref.
ZBP1-PANoptosome	ZBP1 (recognizes Z-DNA/Z-RNA via Zα domain); recruits RIPK3 (via RHIM domain), ASC, NLRP3, Caspase-1, Caspase-8, RIPK1	Viral nucleic acids (Z-form DNA or RNA)	Influenza virus infection; viral infection models	Karki et al. (2022), Lou et al. (2024), Zhan et al. (2024), Li et al. (2025a)
RIPK1-PANoptosome	RIPK1 (forms immunoprecipitation complex with RIPK3, Caspase-8, ASC, FADD, NLRP3)	Bacterial infection-induced signals	*Yersinia* pseudotuberculosis infection	Zu et al. (2021), Mifflin et al. (2020)
AIM2-PANoptosome	AIM2 (recognizes cytosolic DNA); recruits ASC, Caspase-1, Caspase-8, RIPK3, RIPK1, FADD; regulates ZBP1 and pyrin	Cytosolic double-stranded DNA	Herpes simplex virus infection; Francisella novicida infection	Bürckstümmer et al. (2009), Ma et al. (2021)
NLRP12-PANoptosome	NLRP12 (detailed composition incompletely characterized)	Pathogen-associated signals (specific triggers under investigation)	Inflammatory conditions (detailed contexts require further characterization)	Sundaram et al. (2023), Tweedell and Kanneganti (2023)

Abbreviation: ASC, apoptosis-associated speck-like protein containing a caspase recruitment domain; FADD, Fas-associated death domain protein; RHIM, RIP homotypic interaction motif; RIPK, receptor-interacting protein kinase; ZBP1, Z-DNA, binding protein 1.

Although different PANoptosome types respond to distinct stimuli, they share common molecular features. Functionally, PANoptosome components can be categorized into three classes. Sensor molecules such as ZBP1, AIM2, and NLRP3 recognize pathogen-associated molecular patterns (PAMPs) or damage-associated molecular patterns (DAMPs) (Liu K. et al., 2024). Adaptor proteins like ASC and FADD serve as molecular bridges connecting sensors to downstream effector molecules. Catalytic effectors include RIPK1, RIPK3, caspase-1, and caspase-8, whose activation directly executes multiple death pathways. Notably, caspase-6, initially considered an apoptotic executioner molecule, functions as a scaffold in the PANoptosome by enhancing the interaction between RIPK3 and ZBP1 to promote complex assembly (Zheng et al., 2020). These effector molecules, once activated within the PANoptosome complex, execute death through distinct but coordinated mechanisms. Caspase-1 activation leads to gasdermin D cleavage and pyroptosis execution, while promoting maturation and release of pro-inflammatory cytokines IL-1β and IL-18 (Tsuchiya et al., 2019). Caspase-8 activation can both cleave gasdermin D to participate in pyroptosis and activate downstream executioner caspases like caspase-3 to execute apoptosis (Li P.-B. et al., 2024). RIPK3 phosphorylation activates its downstream substrate MLKL, which oligomerizes and translocates to the plasma membrane to form membrane pores, causing cell swelling and rupture (Evans and Coyne, 2019).

Current understanding of PANoptosomes derives predominantly from studies in immune cells, particularly macrophages, and from infection models where the inflammatory death program provides host defense benefits (Sharma et al., 2025; You et al., 2025). Whether analogous molecular complexes form in neurons and glial cells remains incompletely characterized. The neuronal microenvironment differs substantially from immune cells in terms of metabolic state, protein expression profiles, and functional requirements. Neurons may assemble PANoptosomes with distinct composition or regulatory properties, or they may execute multi-pathway death through alternative organizational mechanisms. Additionally, the specific triggers that would induce PANoptosome assembly in the context of metabolic stress and oxidative damage, rather than pathogen-associated molecular patterns, require investigation.

Addressing these questions in cerebral ischemia-reperfusion injury requires examining how multi-pathway death activation occurs across the distinct temporal phases that characterize this condition. Unlike infection models where pathogen recognition provides a discrete triggering event, cerebral ischemia-reperfusion injury unfolds through progressive stages involving initial metabolic failure, subsequent oxidative burst upon blood flow restoration, and prolonged inflammatory amplification. Mitochondria occupy a unique position to coordinate death pathway activation throughout this temporal progression. Their dual role as metabolic engines and death signaling platforms, combined with their sensitivity to both ischemic energy depletion and reperfusion oxidative stress, positions them as potential orchestrators of PANoptosis in this specific pathological context. The following analysis therefore examines how mitochondrial dysfunction evolves temporally and how this evolution might drive coordinated activation of multiple death pathways in cerebral ischemia-reperfusion injury.

## 3 Mitochondrial orchestration of PANoptosis across temporal phases of cerebral ischemia-reperfusion injury

### 3.1 Metabolic crisis and mitochondrial priming during ischemia

Arterial occlusion interrupts blood flow and eliminates both oxygen and glucose delivery to brain tissue with extraordinarily high metabolic demands. ATP synthesis ceases when oxygen becomes unavailable for oxidative phosphorylation. Cellular ATP levels decline precipitously within 3–5 min (Sasaibe et al., 2025; Xu et al., 2025). ATP-dependent ion pumps including sodium-potassium ATPase and calcium ATPase lose function. Sodium accumulates intracellularly while potassium leaks out. Membrane potential depolarizes and opens voltage-gated calcium channels (Akhtar et al., 2025). Glutamate transporters fail and allow excitatory neurotransmitters to accumulate in the extracellular space. NMDA and AMPA receptors become activated and drive additional calcium influx through receptor-operated channels (Amarenco, 2020). Despite these severe perturbations, during ischemia, penumbral regions with residual collateral blood flow experience conditions that prime rather than trigger irreversible death pathway activation when the duration is brief. This distinction between priming and execution explains why penumbral tissue remains salvageable if reperfusion occurs promptly.

Mitochondria attempt to buffer rising cytosolic calcium by importing it through the mitochondrial calcium uniporter. This response initially protects the cytoplasm from calcium-dependent degradative enzymes (Salman, 2018). However, excessive mitochondrial calcium accumulation promotes opening of the mitochondrial permeability transition pore. mPTP opening dissipates membrane potential and creates a cycle where calcium uptake further compromises energetics, which further impairs calcium extrusion, which increases calcium load (Javanmardi et al., 2025; Li Y.-W. et al., 2025). Although oxygen supply is severely limited, residual oxygen molecules remaining in the tissue can still accept leaked electrons from Complex I and Complex III, generating reactive oxygen species even under hypoxic conditions (Feng et al., 2016). This phenomenon occurs because the highly reduced state of the electron transport chain increases the probability of premature electron transfer to available oxygen before reaching Complex IV (Liang et al., 2025). ROS production during ischemia remains modest compared to what will occur during reperfusion, but it begins oxidative damage to mitochondrial proteins, lipids, and mtDNA during ischemia itself. Reduced membrane potential impairs mitochondrial protein import and affects dynamics by allowing fission to proceed while fusion becomes compromised (Han et al., 2022).

Neurons experience these metabolic perturbations most rapidly due to their extraordinary baseline metabolic rates for maintaining synaptic transmission and membrane potential across extensive dendritic and axonal processes (Petersen, 2017; Vardi et al., 2023). Neurons rely almost exclusively on oxidative phosphorylation with minimal glycolytic reserve. High mitochondrial content makes neurons particularly vulnerable when mitochondrial function fails (Huan et al., 2023). In contrast, astrocytes maintain glycogen stores that can be broken down to support anaerobic glycolysis during ischemia. Astrocytes generate ATP without oxygen, though less efficiently, allowing them to maintain ion homeostasis longer than neurons (Stulczewski et al., 2023). This metabolic flexibility explains why astrocytes may undergo reactive transformation rather than death during ischemic conditions that kill adjacent neurons. Neurons also face greater vulnerability to excitotoxicity due to high density of glutamate receptors and active synaptic transmission, while astrocytes express different receptor subtypes and possess more robust calcium buffering (Shi and Lu, 2017; Alcoreza et al., 2021; Yang et al., 2022; Clennell et al., 2023).

The pathological changes described above are initially reversible if reperfusion occurs quickly enough. Mitochondria that have lost some membrane potential can repolarize if oxygen and substrates return. Calcium accumulated in mitochondria can be extruded if ATP becomes available. Moderate oxidative damage can be repaired by antioxidant mechanisms and protein quality control. However, specific thresholds exist beyond which damage becomes irreversible regardless of reperfusion. These thresholds likely correspond to points where MOMP becomes inevitable due to excessive Bcl-2 family protein activation, where calcium triggers permanent mPTP opening, or where oxidative damage destroys critical electron transport chain components beyond repair capacity. The spatial gradient of blood flow deprivation determines how rapidly cells approach these thresholds. In the infarct core where blood flow ceases completely, cells cross irreversibility thresholds rapidly, often within minutes. In contrast, penumbral regions where residual perfusion through collaterals maintains partial oxygen and glucose delivery experience less severe ATP depletion, allowing cells to survive for hours if adequate reperfusion is achieved. Ischemia has thus established conditions for explosive multi-pathway death activation without fully triggering it. Mitochondria have accumulated calcium, suffered oxidative damage, and partially lost membrane integrity. The electron transport chain is highly reduced and primed to generate massive ROS when oxygen suddenly returns.

### 3.2 Mitochondrial collapse and multi-pathway death activation during acute reperfusion

Restoring blood flow paradoxically triggers the most intense phase of injury when oxygen encounters highly reduced electron transport chains. Electrons accumulated during hypoxia transfer to oxygen in an uncontrolled manner and generate superoxide at rates that overwhelm superoxide dismutase capacity (Wolin et al., 2019). Superoxide converts rapidly to hydrogen peroxide, which reacts with transition metals released from damaged proteins to generate hydroxyl radicals through Fenton chemistry (Paradiso et al., 2016; Saudrais et al., 2018; Sirithanakorn and Imlay, 2024). This oxidative burst is not merely collateral damage but an active signaling event that triggers multiple death pathways simultaneously. ROS oxidatively modify NLRP3 inflammasome components and lower the threshold for pyroptotic pathway activation. They stimulate RIPK3 kinase activity and enhance RIPK3-MLKL interactions to facilitate necroptotic execution. This oxidative burst is not merely collateral damage but an active signaling event that triggers multiple death pathways simultaneously. ROS oxidatively modify NLRP3 inflammasome components and lower the threshold for pyroptotic pathway activation (Harijith et al., 2014). They stimulate RIPK3 kinase activity and enhance RIPK3-MLKL interactions to facilitate necroptotic execution (Zhou et al., 2024). Oxidation of cardiolipin, the inner membrane phospholipid anchoring cytochrome c, promotes cytochrome c release and primes the apoptotic cascade (Mulkidjanian et al., 2018; Mahajan et al., 2019; Elmer-Dixon et al., 2020). The oxidative burst enhances calcium channel opening while impairing calcium pump function. This increases calcium influx into already calcium-loaded mitochondria from the ischemic phase. Reciprocally, mitochondrial calcium overload stimulates ROS production by disrupting electron transport chain function. This bidirectional amplification creates a self-reinforcing spiral (Sun et al., 2018). mPTP opening probability increases exponentially when both calcium overload and oxidative stress are present simultaneously. Once mPTP opens, membrane potential collapses completely, ATP synthesis becomes impossible, and mitochondrial matrix contents including mtDNA equilibrate with the cytosol (Guo et al., 2018). Mitochondria swell due to osmotic imbalance. When mPTP opening affects a substantial fraction of cellular mitochondria, cells become committed to death regardless of subsequent interventions.

Oxidative stress and calcium dysregulation converge to trigger mitochondrial outer membrane permeabilization through Bcl-2 family proteins. BH3-only proteins including BID, BIM, and PUMA become activated through p53 activation, endoplasmic reticulum stress, and growth factor withdrawal during ischemia and early reperfusion (Luna-Vargas and Chipuk, 2016; O’Neill et al., 2016; Birkinshaw and Czabotar, 2017). These proteins neutralize anti-apoptotic guardians like Bcl-2 and Bcl-xL. Pro-apoptotic executioners Bax and Bak then oligomerize and insert into the outer membrane to form large pores (Czabotar and Garcia-Saez, 2023). Calcium activates calpains which cleave BID to generate truncated tBID with enhanced pro-apoptotic activity. The convergence of multiple pro-MOMP signals during reperfusion explains why permeabilization occurs broadly and rapidly. Recent evidence suggests that MOMP can contribute to pyroptotic and necroptotic pathway activation as well, though precise mechanisms linking MOMP to these alternative death modes require further investigation (Karch et al., 2015). Both mPTP opening and MOMP facilitate escape of mitochondrial DNA into the cytosol (De Torre-Minguela et al., 2021). Cytosolic mtDNA is recognized as foreign nucleic acid by cGAS. Upon binding double-stranded DNA, cGAS synthesizes cGAMP, which activates STING on the endoplasmic reticulum. STING recruits TBK1, which phosphorylates IRF3 and NF-κB to drive expression of type I interferons and pro-inflammatory cytokines (Hu H. et al., 2022; Wei et al., 2024; Bahat et al., 2025). The inflammatory mediators produced create a cellular environment that promotes inflammasome assembly and pyroptotic pathway engagement.

**FIGURE 2:**
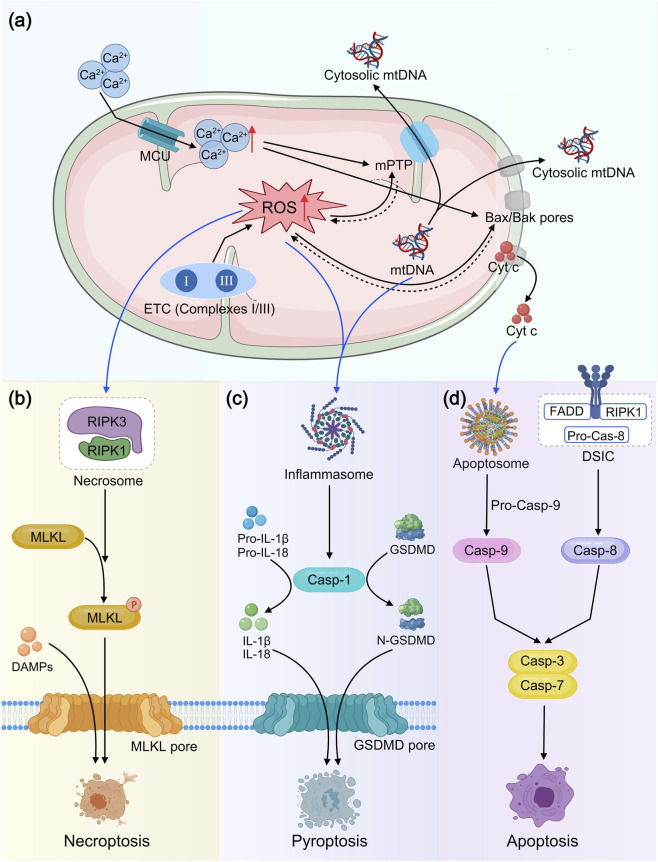
Mitochondrial dysfunction simultaneously triggers necroptotic, apoptotic, and pyroptotic pathways during cerebral ischemia-reperfusion injury. **(a)** Mitochondrial dysfunction hub showing ROS generation, calcium overload, MOMP, and mPTP opening create self-reinforcing cycles that release cytochrome c and mitochondrial DNA. **(b)** Necroptosis pathway where RIPK1/RIPK3 necrosome phosphorylates MLKL to form plasma membrane pores. **(c)** Pyroptosis pathway where inflammasome-activated caspase-1 cleaves gasdermin D and matures inflammatory cytokines. **(d)** Apoptosis pathway where apoptosome and DISC activate caspase-9 and caspase-8 respectively, converging on executioner caspases-3/7. Solid arrows indicate direct activation. Dashed arrows indicate feedback regulation. This figure is created with MedPeer (medpeer.cn).

Emerging evidence suggests that Drp1 may directly participate in death signaling beyond its role in fission through interactions with death receptors and pro-apoptotic proteins (Ruiz et al., 2020; Guo et al., 2021; Tian M. et al., 2025). The molecular architecture of multi-pathway death activation during reperfusion involves concurrent engagement of necroptotic, apoptotic, and pyroptotic machinery. Each pathway is initiated through distinct but convergent signaling cascades. Figure 2 illustrates how mitochondrial dysfunction serves as the central hub orchestrating this concurrent activation. Reperfusion triggers explosive ROS generation from electron transport chain Complexes I and III. Concurrent calcium overload occurs through the mitochondrial calcium uniporter. These events converge to cause mitochondrial outer membrane permeabilization through Bax and Bak oligomerization. They also trigger mitochondrial permeability transition pore opening. MOMP releases cytochrome c into the cytosol. Released cytochrome c binds to APAF-1 to form the apoptosome complex. The apoptosome activates caspase-9, which subsequently activates executioner caspases-3 and caspases-7 to execute apoptosis. Simultaneously, oxidative stress activates RIPK1 and RIPK3 to form the necrosome complex. Activated RIPK3 phosphorylates MLKL. Phosphorylated MLKL oligomerizes and translocates to the plasma membrane to form pores that execute necroptosis. In parallel, released mitochondrial DNA and ROS promote NLRP3 inflammasome assembly. The assembled inflammasome activates caspase-1. Active caspase-1 cleaves gasdermin D to generate pore-forming fragments and processes pro-inflammatory cytokines for pyroptosis execution. The figure demonstrates that these three pathways are activated simultaneously from the common upstream trigger of mitochondrial dysfunction rather than sequentially. Temporal analyses from rodent middle cerebral artery occlusion models demonstrate that activated caspase-3 reaches peak levels between 6 and 24 h post-reperfusion, with the precise timing varying depending on occlusion duration and reperfusion completeness. During this same window, phosphorylated RIPK3 and MLKL appear and colocalize with dying cells (Guida et al., 2017; Speir et al., 2018). Pyroptotic features manifest simultaneously through caspase-1 activation, gasdermin D cleavage, and robust IL-1β and IL-18 release (Li et al., 2017). While the absolute timing may differ across experimental models and species, the critical observation remains consistent: these markers overlap substantially in timing rather than appearing sequentially. Immunofluorescence reveals individual cells expressing activated caspase-3, phosphorylated MLKL, and cleaved gasdermin D simultaneously (Speir et al., 2020; Frank et al., 2022). Electron microscopy shows mixed ultrastructural characteristics with both apoptotic features such as chromatin condensation and necrotic features such as cell swelling and membrane rupture within the same cells (Yan et al., 2022).

Neurons exhibit the most dramatic multi-pathway activation because they experience maximal mitochondrial stress (Stelmashook et al., 2020; Zhu et al., 2024). Their high baseline metabolic demands, near-exclusive dependence on oxidative phosphorylation, and limited glycolytic reserve make them particularly vulnerable when mitochondrial function fails (Hoffmann et al., 2021). The catastrophic mitochondrial collapse during reperfusion triggers concurrent activation of all major death pathways (Demarest et al., 2016; Cheng et al., 2023). Dying neurons release damage-associated molecular patterns including mitochondrial DNA, ATP, and HMGB1 that activate surrounding glial cells and propagate injury signals throughout the ischemic penumbra. Microglia exhibit prominent pyroptotic pathway activation with robust NLRP3 inflammasome assembly, caspase-1 activation, and massive IL-1β and IL-18 secretion (Jekabsone et al., 2023; Cheong et al., 2024; Miao et al., 2025). This reflects their innate immune cell identity and priming to recognize danger signals released from injured neurons. When microglial death occurs under severe oxidative stress or excessive inflammasome activation, pyroptotic membrane rupture releases intracellular contents that create feed-forward inflammatory amplification (Joshi et al., 2019; Wang L. et al., 2024). The inflammatory mediators produced by activated microglia, including TNF-α and IL-1β, sensitize adjacent neurons and astrocytes to death pathway activation, creating spatial propagation of injury beyond the initial ischemic territory. Astrocytes may undergo reactive transformation rather than death under moderate injury conditions due to their capacity for anaerobic glycolysis and robust antioxidant defenses (Fiebig et al., 2019). When ischemia severity exceeds tolerance thresholds, astrocytes undergo death exhibiting multi-pathway features and release DAMPs including ATP, HMGB1, and S100B (Mukda et al., 2019; Wong et al., 2023). These astrocyte-derived danger signals further activate microglia and contribute to progressive barrier dysfunction by stimulating endothelial inflammatory responses. Endothelial cells face oxygen-glucose deprivation during ischemia followed by oxidative stress, hemodynamic shear stress, and inflammatory assault during reperfusion. Their death exhibits mixed apoptotic and necroptotic features with both caspase-3 activation and RIPK3-MLKL pathway engagement detectable in damaged vessels (Liu C. et al., 2025; Yi et al., 2025; Zhao et al., 2025). Endothelial death creates gaps in the blood-brain barrier that dramatically increase permeability and allow plasma protein extravasation, peripheral immune cell infiltration, and potential hemorrhagic transformation (Roth et al., 2025). The coordinated multi-pathway activation observed across these cell types reflects intercellular signaling networks rather than independent cell-autonomous events. Death of one cell type amplifies injury in neighboring cells through DAMP release and inflammatory mediator production.

### 3.3 Impaired mitochondrial quality control and sustained inflammatory amplification

Damaged mitochondria should be identified by quality control mechanisms and selectively removed through mitophagy before triggering cell death programs (Sun et al., 2022; Gross, 2024; Yang et al., 2024). The PINK1-Parkin pathway normally mediates this clearance. When mitochondria lose membrane potential, PINK1 import is blocked and PINK1 accumulates on the outer membrane where it phosphorylates ubiquitin and recruits Parkin. Activated Parkin ubiquitinates outer membrane proteins and creates recognition signals for autophagy receptors including p62 and optineurin, which recruit autophagosome assembly machinery (Narendra and Youle, 2024). Alternative pathways operate through receptor proteins including BNIP3, NIX, and FUNDC1, which directly bind autophagy proteins without requiring ubiquitination (Nguyen-Dien et al., 2023; Sun et al., 2024; Yamashita et al., 2024). However, the magnitude and rapidity of mitochondrial damage during cerebral ischemia-reperfusion injury overwhelms these protective mechanisms. Even if mitophagy machinery were functioning at full capacity, the rate of damage generation during reperfusion would vastly outpace clearance (Cui et al., 2022; Zhang et al., 2022; Li Y. et al., 2025). Moreover, ischemia-reperfusion directly impairs autophagy function, further compromising this protective response. Energy depletion limits ATP-dependent steps in autophagosome formation. Oxidative stress damages lysosomal membranes and compromises their degradative capacity (Jiao et al., 2024). Calcium dysregulation disrupts autophagy signaling pathways. Excessive mitochondrial fragmentation creates overwhelming numbers of potential targets that saturate the autophagy machinery. The combination of massive damage generation and impaired clearance results in accumulation of damaged mitochondrial fragments (Ma et al., 2020; Ma et al., 2025; Chen et al., 2024).

Damaged mitochondria that escape clearance become persistent sources of danger signals over days to weeks following acute reperfusion. This sustained danger signal emission maintains death pathway activation at levels insufficient to immediately kill cells but sufficient to keep them chronically stressed. Death receptor pathways remain sensitized (Glover et al., 2024; Hall-Younger and Tait, 2025). Cells in this chronically stressed state eventually undergo death, and because death pathways remain engaged, this death exhibits PANoptosis features with lytic membrane rupture releasing cellular contents into the extracellular space (Almars et al., 2025; Wang Y. et al., 2025). Multiple DAMPs are released from dying cells and each engages distinct receptors on surrounding cells. HMGB1 binds TLR4 and RAGE on microglia and astrocytes to trigger NF-κB activation and inflammatory cytokine production (Shi et al., 2018). Mitochondrial DNA activates cGAS-STING pathways in recipient cells and TLR9 on immune cells (Cho and Hsieh, 2016). S100 proteins from damaged astrocytes amplify inflammatory signaling (Yao et al., 2016). Mature IL-1β and IL-18 from dying cells induce adhesion molecule expression on endothelial cells and stimulate additional inflammatory mediator production from activated glia. Released danger signals activate surrounding microglia and astrocytes to produce TNF-α, IL-6, IL-1β, chemokines, and reactive oxygen species (Lee et al., 2022; Wang C. et al., 2024). These mediators damage adjacent cells not directly injured during initial ischemia or acute reperfusion. When these secondarily injured cells undergo PANoptosis, they release additional DAMPs and propagate inflammatory waves outward from the infarct core into previously viable tissue. This self-perpetuating cycle explains why infarct volumes expand over hours to days following reperfusion even when blood flow has been successfully restored.

Endothelial cell death causes progressive blood-brain barrier breakdown over days. Increased permeability allows extravasation of plasma proteins including albumin, fibrinogen, and complement components, which promote edema formation and exert neurotoxic effects (Wang S. et al., 2024; Tian Y. et al., 2025). Vasogenic edema increases intracranial pressure and causes secondary injury through mechanical compression. Barrier disruption permits infiltration of peripheral immune cells (Zhou et al., 2021; Liu G. et al., 2025). Neutrophils arrive within hours and release matrix metalloproteinases that further degrade barrier integrity, proteases that damage extracellular matrix, and oxidants that injure surrounding tissue (Hung et al., 2020; Nowaczewska-Kuchta et al., 2025). Monocytes infiltrate over subsequent days and differentiate into macrophages. Lymphocytes arrive later and contribute through cytotoxic mechanisms and cytokine production (Qiu et al., 2021; Xie and Li, 2021). This sequential recruitment transforms localized neuronal death into widespread inflammatory pathology involving multiple cell types and peripheral immune components. The temporal extension over days provides a wider therapeutic window for interventions targeting this phase. This window is wider than the narrow window for preventing acute mitochondrial collapse. This sustained inflammation phase represents a distinct therapeutic opportunity. Interventions can target danger signal neutralization and clearance-enhancing strategies rather than direct pathway inhibition. Examples include autophagy enhancers that improve clearance if appropriately timed. DAMP neutralization approaches include HMGB1 antibodies and TLR4 antagonists. Other examples are inflammasome inhibitors targeting NLRP3 or caspase-1 and agents promoting blood-brain barrier repair (Koide et al., 2023; Wang et al., 2023; Liu L. et al., 2025). Evidence for these approaches remains primarily preclinical. However, the wider therapeutic window makes them more practically feasible than interventions requiring treatment within minutes of stroke onset.

### 3.4 Integration and evidence for PANoptosis in cerebral ischemia-reperfusion injury

The preceding subsections described how ischemia primes mitochondrial dysfunction, how reperfusion triggers explosive multi-pathway activation, and how impaired quality control sustains death signaling over time. This temporal progression raises the question of whether cerebral ischemia-reperfusion injury involves PANoptosis or simply reflects independent parallel activation of distinct death pathways. Weighing this question requires examining multiple lines of evidence rather than relying on a single experimental approach. Four categories of evidence merit consideration for determining whether the observed phenomena align with PANoptosis characteristics.

**FIGURE 3:**
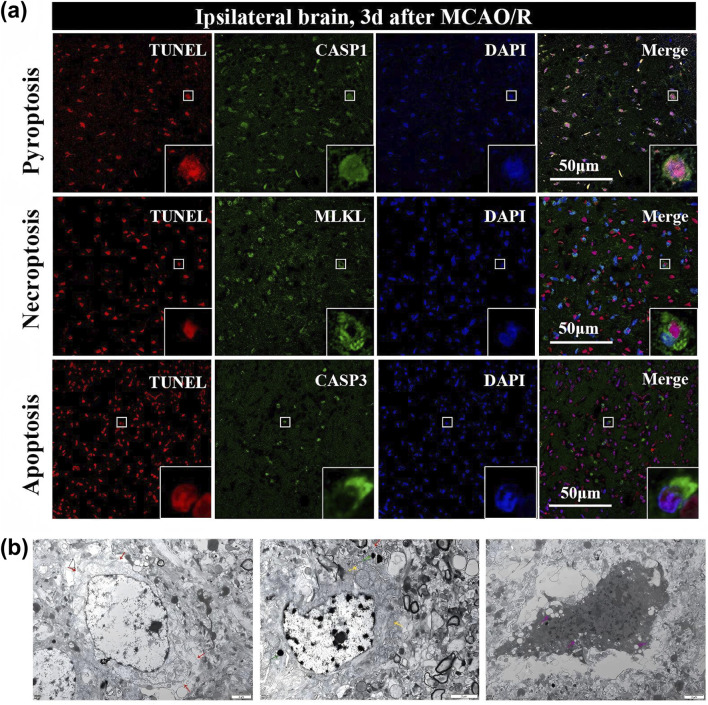
Multi-pathway death activation in MCAO/R models [155]. **(a)** Immunofluorescence co-localization of TUNEL with CASP1, MLKL, and CASP3 in individual cells. **(b)** Transmission electron microscopy showing mixed morphological features in dying neurons. Copyright 2025, Elsevier.

Temporal evidence demonstrates that markers of apoptotic, necroptotic, and pyroptotic pathways appear during overlapping time windows following reperfusion rather than in sequence (Yan et al., 2022). Activated caspase-3, phosphorylated RIPK3 and MLKL, activated caspase-1, and cleaved gasdermin D all reach peak levels within the first 24 h post-reperfusion with substantial temporal overlap. Studies from independent laboratories in different countries consistently report this concurrent appearance pattern. The temporal overlap argues against sequential activation where cells commit to one pathway then switch to another. This concurrent appearance is more consistent with simultaneous pathway engagement than with sequential activation where cells commit to one pathway then switch to another. Morphological evidence reveals individual cells simultaneously displaying features of multiple death modalities (She et al., 2023). Electron microscopy shows mixed ultrastructural characteristics combining apoptotic features such as chromatin condensation with necrotic features such as cell swelling and membrane rupture within the same cells (Cheng et al., 2024). Immunofluorescence demonstrates single neurons co-expressing activated caspase-3, phosphorylated MLKL, and cleaved gasdermin D. Advanced imaging techniques including multiplexed immunofluorescence confirm that individual dying cells express markers from all three pathways simultaneously. Direct visualization in ischemic brain tissue demonstrates concurrent activation of multiple death pathways within individual cells, with co-expression of pyroptotic, necroptotic, and apoptotic markers and mixed ultrastructural features (Figure 3). This phenotypic mixing at the single-cell level provides strong morphological evidence for integrated rather than independent death pathway activation (Zhang et al., 2024). Functional evidence from pathway inhibition studies shows that blockade of individual death pathways provides incomplete neuroprotection. Across different experimental conditions, the consistent pattern is that alternative pathways remain functional when one pathway is blocked. If cells committed exclusively to single pathways, complete blockade of a given pathway should completely protect cells attempting to use it. The pattern of partial protection, observed across diverse experimental conditions, indicates that alternative pathways remain functional and can execute death when one pathway is blocked. Preliminary evidence suggests that combining interventions targeting multiple pathways may provide additive protection (Tang et al., 2025; Wang L. et al., 2025).

Mechanistic evidence demonstrates how mitochondrial dysfunction simultaneously triggers molecular events associated with all three major death pathways. MOMP releases factors engaging apoptotic machinery while potentially contributing to other pathways. mtDNA activates cGAS-STING and creates inflammatory conditions promoting pyroptosis while upregulating necroptotic components (Wu et al., 2025). ROS directly modifies NLRP3, RIPK3, and pro-apoptotic Bcl-2 family members. Calcium overload activates calpains that process multiple death pathway substrates. The kinetics of mitochondrial dysfunction match the kinetics of multi-pathway activation. Peak mitochondrial ROS generation and calcium dysregulation correspond to the onset of concurrent caspase-3, RIPK3-MLKL, and caspase-1 activation. Mitochondrial-targeted interventions reduce markers across all three pathways simultaneously. This supports mitochondrial dysfunction as a coordinating mechanism. The convergence of multiple mechanisms on simultaneous multi-pathway activation provides mechanistic plausibility for PANoptosis occurrence.

The extensive evidence for concurrent multi-pathway activation, phenotypic mixing, pathway redundancy, and mitochondrial convergence strongly suggests coordinated multi-pathway death in cerebral ischemia-reperfusion injury. This pattern contrasts with mutually exclusive single-pathway commitments. Table 2 summarizes how mitochondrial dysfunction evolves through three distinct temporal phases. Each phase shows different patterns of PANoptosis activation and therapeutic accessibility. During ischemic priming, mitochondria accumulate damage without triggering irreversible death commitment. This creates a narrow window for reperfusion therapy. Acute reperfusion triggers explosive mitochondrial collapse with concurrent multi-pathway activation. This pattern is evident from co-expression of pathway markers in individual cells and incomplete protection by single-pathway inhibition. Sustained inflammation emerges when failed mitochondrial clearance drives chronic inflammatory amplification. This phase offers an extended therapeutic window for interventions that target danger signal neutralization and autophagy enhancement. The PANoptosis framework explains several observations that are difficult to account for under the traditional model of independent death pathways. It explains why dying cells exhibit mixed morphological features rather than pure apoptotic, necroptotic, or pyroptotic phenotypes. It explains why single-pathway inhibitors consistently provide incomplete protection across diverse preclinical models. It explains why interventions targeting mitochondrial dysfunction can reduce markers of multiple death pathways simultaneously. It explains why the inflammatory response appears robust despite cells presumably attempting to die through non-inflammatory apoptotic pathways. Most importantly for therapeutic development, the PANoptosis framework suggests that effective neuroprotection may require addressing pathway redundancy through multi-target approaches or by targeting upstream convergence points like mitochondrial dysfunction.

**TABLE 2:** Temporal phases of mitochondrial-mediated PANoptosis in cerebral ischemia-reperfusion injury.

Phase	Duration	Primary mitochondrial dysfunction	PANoptosis activation pattern	Defining characteristics	Therapeutic window
Ischemic priming	Minutes to hours during occlusion	Energy depletion and calcium accumulation without irreversible membrane damage	Pathway priming: Accumulation of death machinery components without execution	Reversible dysfunction; cells remain viable if reperfused promptly	Very narrow; requires immediate reperfusion
Acute reperfusion	First 24 h after flow restoration	Oxidative burst with membrane permeabilization and mitochondrial DNA release	Concurrent activation: simultaneous engagement of apoptotic, necroptotic, and pyroptotic pathways	Co-expression of pathway markers in individual cells; mixed morphological features; incomplete protection by single-pathway inhibition	Narrow; hours for direct pathway intervention
Sustained inflammation	Days to weeks post-injury	Failed clearance of damaged mitochondria with persistent danger signal emission	Chronic activation: sustained low-level multi-pathway engagement driving inflammatory amplification	Progressive secondary injury; DAMP-mediated feed-forward loops; peripheral immune infiltration	Extended; days for anti-inflammatory and clearance-enhancing strategies

Abbreviations: DAMP, damage-associated molecular pattern; DNA, deoxyribonucleic acid.

## 4 Current challenges and future directions

### 4.1 Critical research gaps and technical challenges

The most significant limitation in current evidence is the absence of direct demonstration of PANoptosome complex assembly in brain tissue from ischemia-reperfusion injury models. In infection models, researchers have used co-immunoprecipitation to pull down protein complexes containing RIPK1, RIPK3, caspase-8, caspase-1, ASC, NLRP3, and FADD simultaneously (Place et al., 2021; Cheng et al., 2025). Proximity ligation assays visualize protein-protein interactions in tissue sections (Liu L. et al., 2024). However, these techniques have not successfully demonstrated PANoptosome formation in ischemic brain tissue. PANoptosomes are large but transient complexes that may exist in only a subset of dying cells at any given moment. They require fresh or carefully preserved tissue for detection. Brain tissue presents unique challenges for biochemical extraction and antibody penetration compared to cultured cells or peripheral immune tissues.

Animal models possess inherent limitations that constrain translational relevance. Rodent middle cerebral artery occlusion models use young healthy animals without comorbidities, whereas human stroke patients typically present with advanced age, hypertension, diabetes, and hyperlipidemia that profoundly influence cellular stress responses (Martha et al., 2018; McPherson et al., 2018). Experimental protocols employ standardized occlusion durations and reperfusion timing that differ from heterogeneous clinical scenarios where ischemia duration varies widely and reperfusion may be incomplete or delayed by hours. Collateral circulation varies substantially between mouse strains and does not fully replicate human vascular anatomy. Collectively, these differences between experimental models and clinical reality may explain why numerous neuroprotective strategies effective in rodents have failed in clinical trials. Direct evidence from human brain tissue remains extremely limited due to the scarcity of acute ischemic samples suitable for molecular analysis. While indirect evidence from cerebrospinal fluid biomarkers and postmortem studies suggests multi-pathway activation in human stroke patients, definitive demonstration of PANoptosome formation in human neurons requires fresh surgical specimens that are rarely available. Cell-type-specific interrogation of death pathway activation requires technical approaches beyond traditional histology. Traditional approaches, including bulk tissue analyses, detect population-average changes but cannot resolve which cell types undergo which forms of death at single-cell resolution. Multiplexed immunofluorescence enables simultaneous detection of multiple markers while preserving spatial context and has shown promise for visualizing concurrent expression of death pathway markers in individual cells (Parra, 2018; Cooper et al., 2021; Wu et al., 2022). Imaging mass cytometry extends multiplexing capacity to dozens of proteins at single-cell resolution. However, these technologies remain expensive and technically demanding. Standardized protocols optimized for brain tissue and validated antibody panels are still under development.

### 4.2 Translational barriers and therapeutic development strategies

The therapeutic time window represents the most important challenge separating experimental findings from clinical applications. Patients typically arrive at hospitals hours after symptom onset, long after the metabolic failure that occurs during ischemia has initiated and potentially after irreversible death pathway commitment. The median time from symptom onset to hospital arrival exceeds 3 h in most stroke registries (Davey et al., 2018). This clinical reality makes interventions targeting mitochondrial dysfunction during ischemia or acute reperfusion difficult to implement except in specialized scenarios such as elective surgeries or as adjunctive therapy during endovascular thrombectomy. The inflammatory amplification phase driven by DAMP release extends over hours to days following acute reperfusion and potentially provides a wider therapeutic window for interventions targeting downstream consequences rather than initial death pathway activation (Yu S. et al., 2021). Blood-brain barrier penetration poses another obstacle, as many candidate therapeutic molecules exhibit poor central nervous system entry. Although barrier disruption during cerebral ischemia-reperfusion injury temporarily increases permeability, this disruption is spatially and temporally heterogeneous. Nanoparticle carriers, receptor-targeting strategies, and intranasal administration represent potential solutions requiring extensive development to optimize formulation and establish safety profiles (Saito et al., 2016; Liu et al., 2022; Liu et al., 2024 Y.; Lu et al., 2022).

Upstream interventions directed at mitochondrial protection target the initial stages of injury during ischemia or acute reperfusion and could prevent activation of multiple death pathways simultaneously. Mitochondria-targeted antioxidants, and mitochondrial dynamics modulators address pathway redundancy more effectively than single-pathway inhibitors by preventing the upstream mitochondrial dysfunction that drives multiple death pathways simultaneously, rather than blocking individual downstream executioners while leaving alternative routes functional (Isaev et al., 2016; Maneechote et al., 2017). However, they face challenges including potential off-target effects and the fundamental problem that mitochondrial function is essential for cell survival. Downstream interventions targeting specific PANoptosis components offer greater molecular specificity but encounter pathway redundancy where blocking one component may shift death execution to alternative pathways. Combination therapy targeting multiple points simultaneously represents a logical response. Potential combinations include pairing mitochondrial protection with caspase inhibition or combining antioxidants with RIPK1 inhibitors and anti-inflammatory drugs. Preliminary preclinical evidence suggests that combined interventions may provide additive protection exceeding what any single intervention achieves (Xu et al., 2018; Amarenco et al., 2021). However, combination approaches introduce complexity regarding optimal drug ratios, potential drug interactions, additive toxicities, and substantially increased development costs. Addressing inflammatory amplification and enhancing damaged mitochondria clearance offer alternative strategies that may circumvent some limitations of acute death pathway blockade and provide benefit even when administered hours after ischemia onset.

## 5 Conclusion

Evidence from cerebral ischemia-reperfusion injury supports coordinated multi-pathway cell death consistent with PANoptosis characteristics. Mitochondrial dysfunction represents the convergence point where outer membrane permeabilization, mitochondrial DNA release, reactive oxygen species generation, calcium dysregulation, dynamics imbalance, and autophagy deficiency simultaneously activate apoptotic, necroptotic, and pyroptotic pathways. Ischemia primes mitochondria through ATP depletion and calcium accumulation without immediately triggering irreversible death. Reperfusion causes explosive mitochondrial collapse when oxygen encounters reduced electron transport chains, generating massive oxidative stress that triggers concurrent multi-pathway activation within hours. Impaired quality control allows damaged mitochondria to accumulate and persistently emit danger signals, driving inflammatory amplification over days to weeks. Temporal co-occurrence of pathway markers, mixed morphological features in individual cells, incomplete protection from single-pathway inhibition, and mechanistic evidence for mitochondrial convergence support the PANoptosis framework. Direct PANoptosome detection during cerebral ischemia-reperfusion injury remains an important technical challenge. This framework clarifies why single-pathway blockade provides only partial neuroprotection and why clinical translation has proven difficult. Therapeutic strategies may require targeting upstream mitochondrial dysfunction or addressing pathway redundancy through multi-target approaches. Sustained inflammatory amplification offers wider intervention windows than acute mitochondrial collapse, potentially enabling more clinically feasible treatment strategies.
